# Evaluation of Anticancer Potential of *Ganoderma lucidum* on MCF-7 Breast Cancer Cells Through Genetic Transcription of Energy Metabolism

**DOI:** 10.3390/antiox14121471

**Published:** 2025-12-08

**Authors:** Levent Gülüm, Emrah Güler, Emir Çapkınoğlu, Ayşe Büşranur Çelik, Yusuf Tutar

**Affiliations:** 1 Department of Plant and Animal Production, Mudurnu Süreyya Astarcı Vocational College, Bolu Abant İzzet Baysal University, Bolu 14030, Turkey; leventgulum@ibu.edu.tr; 2 Medicinal and Aromatic Plants Research Group, Innovative Food Technologies Development Application and Research Center, Bolu Abant Izzet Baysal University, Bolu 14030, Turkey; 3 Department of Horticulture, Faculty of Agriculture, Bolu Abant İzzet Baysal University, Bolu 14030, Turkey; emrahguler6@gmail.com; 4 Department of General Surgery, School of Medicine, Acibadem Mehmet Ali Aydinlar University, Istanbul 34638, Turkey; 5 Molecular Biology and Genetics, University of Health Sciences, Istanbul 34668, Turkey; ayseclk1899@gmail.com; 6 Department of Basic Medical Sciences, Division of Medical Biochemistry, Faculty of Medicine, Recep Tayyip Erdogan University, Rize 53020, Turkey; yusuf.tutar@erdogan.edu.tr; 7 Department of Basic Pharmaceutical Sciences, Division of Biochemistry, Faculty of Pharmacy, University of Health Sciences, Istanbul 34668, Turkey

**Keywords:** *Ganoderma lucidum*, anticancer, volatile compounds, energy metabolism, Lingzhi, MCF-7 cells, doxorubicin

## Abstract

*Ganoderma lucidum* has long been recognized for its medicinal properties, particularly due to its antioxidant, anti-inflammatory, and pro-apoptotic components such as polysaccharides and triterpenoids. This study aimed to evaluate the cytotoxic and molecular effects of ethanol and methanol extracts of *G. lucidum* as well as doxorubicin on MCF-7 breast cancer cells. The cytotoxicity was assessed via MTT assay. The methanol extract showed stronger cytotoxicity (IC_50_: 62.37 µg/mL) than the ethanol extract, while doxorubicin exhibited an IC_50_ value of 0.66 mM. Phenolic profiling by HPLC revealed high levels of vanillic acid, gallic acid and (−)-epicatechin in the methanol extract, while volatile compounds such as hexanal and acetic acid were identified by GC-MS. Flow cytometric analysis demonstrated G0/G1 phase cell cycle arrest and an increase in early and late apoptotic populations. Gene expression studies using RT-qPCR showed significant downregulation of *ACAT1*, *ADCY3*, and *NME2,* key regulators of energy metabolism and epigenetic modification. On the other hand, doxorubicin treatment upregulated *ACAT1* and *ADCY3*, while a slight downregulation was observed in *NME2*. These molecular changes suggest that *G. lucidum* induces apoptosis and impairs cancer cell proliferation through metabolic disruption and gene modulation.

## 1. Introduction

Breast cancer is a significant global health issue and is the most diagnosed cancer worldwide. In 2020, there were approximately 2.3 million new cases, which accounted for 30% of all female cancers and 11.7% of all cancer cases, surpassing lung cancer in incidence [1,2]. Breast cancer is the second leading cause of cancer-related deaths among women, following lung cancer [2,3], with an estimated 685,000 deaths reported globally in 2020 [3,4]. Natural compounds have attracted considerable attention in cancer research due to their multi-targeted mechanisms and more favorable safety profiles compared to conventional therapies. These agents exhibit anticancer effects by inducing apoptosis, modulating the cell cycle, and inhibiting metastasis. Ferulic acid, for example, modulates the activity of tissue transglutaminase and impacts pathways associated with reactive oxygen species (ROS) [5,6]. Additionally, phytochemicals help regulate oxidative stress, which limits the proliferation of malignant cells [7,8], and they demonstrate anti-metastatic activity in breast cancer models [9]. Their effects on cell signaling and metabolism highlight their therapeutic potential [10,11]. Epidemiological studies have linked a high dietary intake of phytochemicals with a reduced risk of breast cancer [12]. Furthermore, combinations of compounds such as curcumin, epicatechin, and thymoquinone have been shown to enhance responses to conventional treatments [13,14]. Natural antioxidants from sources like *Sargassum polycystum* and milk thistle (silibinin) also provide additional anticancer effects [15,16,17]. With generally lower toxicity, these compounds are promising candidates for integrative cancer therapy. However, further clinical trials are needed to confirm their efficacy and safety [11,18]. 

*Ganoderma lucidum* (Curtis) P. Karst (*Ganodermataceae*), commonly known as Reishi or Lingzhi [19], is widely recognized for its various pharmacological properties thanks to its high bioactive pyhtochemicals such as phenolics and volatile aromatic compounds. Among the various phenolic compounds with anticancer potential, ferulic acid is one of the most extensively studied, demonstrating a remarkable ability to induce apoptosis and inhibit cell proliferation across various cancer cell lines by regulating key signaling pathways like PI3K/Akt and ERK [20,21,22]. Beyond its direct cytotoxic effects, FA also mitigates the tumor microenvironment by suppressing inflammatory markers and preventing oxidative stress-related progression [23,24]. Other compounds also show promise; Vanillic acid, for instance, exhibits moderate anticancer activity through its antioxidant properties and its ability to downregulate cancer-related pathways [25]. Procyanidins, a class of flavonoids, contribute by inducing apoptosis and have been noted for their potential to enhance the efficacy of chemotherapeutic agents, suggesting a role in adjunctive therapy [26,27]. Recent research highlights succinic cid and syringin’s potential to impact cancer cell metabolism and induce apoptosis, though further investigation is needed to clarify their mechanisms [28].

The potential anticancer properties of several volatile and low-molecular-weight compounds commonly found in plant-derived extracts, such as hexanal and nonanal, have been associated with cytotoxic and antioxidant activities that may contribute to the inhibition of cancer cell proliferation, particularly in plant species traditionally used for cancer treatment [29,30]. Benzaldehydes have demonstrated therapeutic potential by inducing apoptosis and oxidative stress in cancer cells, suggesting its involvement in pathways regulating cell death and tumor suppression [31]. 1-pentanol has exhibited inhibitory effects on cellular growth, implying a possible role in modulating cancer cell metabolism [32]. The 6-methyl-5-hepten-2-one, a naturally occurring ketone, has been reported to exert notable antioxidant capacity [33]. Additionally, acetic acid and its derivatives—such as 2-pyridinepropanoic acid ethyl ester—have shown biological activities including pro-apoptotic and antiproliferative effects, suggesting their relevance as potential natural agents in cancer therapy [34,35].

The investigation of metabolic genes in the breast cancer is crucial for understanding the complexities of metabolic reprogramming and its implications for cancer progression and therapy. ACAT1 is a key enzyme in lipid metabolism that promotes the use of acetate for lipid synthesis, which is crucial for building new cell membranes in rapidly proliferating cancer cells and thus facilitates tumor growth [36,37]. ADCY3 regulates cAMP signaling, and its upregulation in breast cancer enhances glycolysis, promoting the Warburg effect to support increased cell proliferation [38]. NME2 is integral to nucleotide metabolism and cellular energy homeostasis, and its expression is inversely correlated with metastatic potential, making it a critical factor in tumor progression and a potential therapeutic target [37,39,40].

Although *G. lucidum* is well-known for its antioxidant and anticancer properties, its molecular effects on hormone receptor-positive breast cancer cells are not yet fully understood. This study aimed to evaluate the cytotoxic activity of various extracts on MCF-7 cells, assess the effectiveness of different solvents, and characterize the phytochemical composition of the extracts. This research addresses a gap in literature by examining the functional impacts on cell cycle regulation and apoptosis through a gene-level analysis. In particular, this study evaluated the anticancer potential of the methanolic extract of *G. lucidum* on MCF-7 human breast cancer cells by examining the expression of energy metabolism genes for the first time.

## 2. Material and Methods

### 2.1. Mushroom Material and Extract Preparation

**Figure 1:**
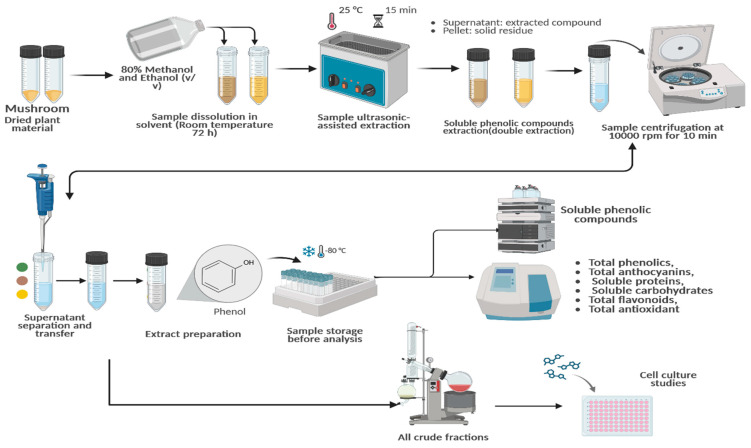
Flowchart detailing the extraction of *G. lucidum*, the identification of bioactive compounds, and subsequent cellular studies. https://BioRender.com/t0r0hnm, Created in BioRender. TUTAR, Y. (2025) https://BioRender.com/t0r0hnm. Access date: 12 June 2025.

Samples of *G. lucidum* were obtained from the Department of Horticulture at Bolu Abant İzzet Baysal University, where they were cultivated by “Nice Tarım ^®^.” The mycelia were purchased from commercial suppliers, and the mushrooms were grown in the faculty’s mushroom farming chamber. Extraction was performed by modifications to the method of Mousavi et al. [41]. The dried and powdered samples (2 g) were dissolved in 25 mL of solvent (80% ethanol and 80% methanol) This mixture was left at room temperature for 72 h. After the incubation period, the mixture was sonicated for 15 min using an ultrasound device from HY Technologies (Cairo, Egypt). The solution was then filtered through Whatman No. 1 filter paper, and the sonication process was repeated with an equal volume of fresh solvent to ensure maximum extraction efficiency. Following sonication, the combined extracts were centrifuged at 10,000 rpm for 10 min. These extracts were analyzed for their total content of phenolics, anthocyanins, proteins, carbohydrates, and flavonoids, as well as antioxidant properties. For cytotoxicity studies, the resulting supernatants were concentrated under vacuum using a rotary evaporator, and all crude fractions were stored at −20 °C. The extracts were then dissolved in dimethyl sulfoxide (DMSO) to prepare stock solutions at a concentration of 1 mg/mL. Required concentrations for the experiments were subsequently prepared from this stock solution (Figure 1).

### 2.2. Phytochemical Measurements

#### 2.2.1. Total Phenolics (TPC)

To determine TPC, we utilized a modified microscale method based on prior research [42,43], using a modified Folin–Ciocalteu method. First, 100 µL of the sample was mixed with 1600 µL of distilled water and 50 µL of Folin–Ciocalteu reagent. The mixture was vortexed and 300 µL of a 7% sodium carbonate solution was added, and the mixture was vortexed again. After incubating in the dark for two hours, the absorbance was measured at 760 nm. Gallic acid, serially diluted from 2 mM seven times, was used as the standard, and the results were expressed as millimoles (mM) of gallic acid equivalents (GAE) per gram dry weight (DW) of *G. lucidum* sample.

#### 2.2.2. Total Flavonoids and Anthocyanins

The total flavonoid content (TFC) was measured using a modified colorimetric method. During this process, the mushroom extracts or standards were reacted with sodium nitrite, aluminum chloride, and sodium hydroxide as reported in [31]. Then, the absorbance was measured at 430 nm. Quercetin was used as the standard, and the results were expressed in mM in DW [44].

To determine the total anthocyanin content, the sample was mixed with a solution of 30% ethanol and 1% hydrochloric acid (HCl). Then, the mixture was measured spectrophotometrically at 540 nm. The results were calculated as mM of malvidin-3-glucoside equivalents in DW using the following formula:

Tant = 16.7 × A540 × Df

In this formula, Df represents the dilution factor [45].

#### 2.2.3. Soluble Proteins and Carbohydrates Analysis

The total soluble protein content was determined using a modified Bradford method. For this analysis, 100 µL of the sample was mixed with 1.9 mL of Bradford reagent, and the absorbance was measured at 595 nm using bovine serum albumin (BSA) as the standard. The total carbohydrate content was analyzed using a modified phenol-sulfuric acid method. For this analysis, 100 µL of the sample was treated with diluted phenol and concentrated sulfuric acid. Absorbance was measured using glucose standards, and the results were expressed as a percentage [46].

#### 2.2.4. Total Antioxidant Capacity Analysis

The DPPH radical scavenging activity was assessed using a previously reported method [47]. DPPH was dissolved in ethanol to achieve an absorbance of 700–800 at 517 nm. Then, 1900 µL of the solution was combined with 100 µL of the sample or ascorbic acid standard. After incubating for 15 min, the absorbance was measured spectrophotometrically. Results were calculated using the ascorbic acid calibration curve and expressed in mM.

Copper (II) ion reducing antioxidant capacity (CUPRAC) was analyzed using a modified method based on one developed by Apak et al. [48] for copper (II) ion reduction. For the analysis, a reagent consisting of a 10 mM copper (II) solution, a 7.5 mM neocuproine (Nc) solution, and a 1 M ammonium acetate (NH_4_Ac) solution was prepared. Next, 1900 µL of this reagent were added to 100 µL of the sample. The mixture was vortexed and allowed to stand at room temperature for 20 min. As a standard, ascorbic acid was serially diluted from a concentration of 2 mM and treated using the above-described method. The absorbance of the prepared samples and standards was measured with a spectrophotometer at a wavelength of 450 nm, and the results were expressed in mM.

The ABTS^•+^ radical scavenging activity was measured using a modified method based on that of Bulut et al. [49]. First, a 7 mM ABTS solution with potassium persulfate was incubated in the dark for 12–16 h to generate radicals. Then, the solution was diluted with sodium acetate buffer (pH 4.5) until the absorbance reached 0.7 ± 0.01 at 734 nm. Then, samples and ascorbic acid standards were added to the ABTS solution and incubated for 15 min at room temperature. Antioxidant activity was calculated using the ascorbic acid standard curve and expressed in mM.

Ferric (III) Reducing Antioxidant Power (FRAP) procedure was followed in the study of [50]. The reagent was diluted at 1:1 for use in the analysis. The assay comprised 100 microliters of both the sample and standard solution, combined with 1900 microliters of FRAP reagent. 2 mM solution of L-ascorbic acid was utilized as a standard through serial dilution. The concentrations of the samples were calculated using the equation derived from the L-ascorbic acid standards and expressed in mM.

### 2.3. Phenolic Compound Profiling by HPLC

The HPLC separation method was used to determine phenolic compounds according to the procedure by Rodriguez-Delgado et al. [51]. Phenolic compound analysis was conducted using a Waters HPLC system, which included dual 510 pumps, an automated gradient controller (Model 680), a Rheodyne 7125 injector with a 20 μL loop, a programmable fluorescence detector (Model 470), and a tunable absorbance detector (Model 486) (Waters Corporation, Milford, MA, USA). The chromatographic data was processed using Baseline Workstation 810 software. The analytical column utilized was a Nova-Pak C18 (150 mm × 3.9 mm I.D., 4 μm particle size), accompanied by a matching guard column. Peak purity was confirmed with a Beckman 168 diode array detector, where purity was defined as a spectral match factor of ≥99.5%. Separation was achieved through gradient elution using mobile phase A (methanol–acetic acid–water in a ratio of 10:2:88, *v*/*v*) and mobile phase B (methanol–acetic acid–water in a ratio of 90:2:8, *v*/*v*), with a total flow rate set at 1.0 mL/min. Absorbance was monitored at 280 nm, while fluorescence detection parameters were set at λ_ex = 278 nm/λ_em = 360 nm and λ_ex = 330 nm/λ_em = 374 nm, with a bandwidth of ±2 nm.

Sample Preparation and Quantification; Analytical standards, including gallic acid, 4-aminobenzoic acid, procatechin, chlorogenic acid, syringic acid, 4-hydroxybenzoic acid, syringrin hydrate, caffeic acid, vanillic acid, ferulic, sinapic acid, coumaric acid, rutintrihydrate, quercitrin, (−)-epicatechin, (+)-catechin, salicylic acid, succinic acid were sourced from Sigma-Aldrich (Darmstadt, Germany) and prepared in a matrix solution of methanol. Standards were prepared by serial dilution of 80 mg/L six times and were stored at −4 °C in the dark. All samples were filtered through 0.45 µm cellulose acetate membranes before injection. Quantification was conducted using internal standardization with 2,5-dihydroxybenzaldehyde at a concentration of 34.4 mg/L, and peak identification was based on comparisons with authentic standards (Figure S1). Standard curves were created by a linear regression analysis (R^2^ > 0.99), and contents were calculated using the equation obtained from the curves.

### 2.4. Determination of Volatile Compounds

For the determination of volatile compounds, minor modifications were made to the method of Wang et al. [52]. The volatile compounds were analyzed using a Shimadzu GCMS-QP2010 (Shimadzu Corporation, Kyoto, Japan) equipped with an Rxi-5ms fused-silica capillary column (60 m × 0.25 mm × 0.25 μm, 5% diphenyl/95% dimethyl polysiloxane; Restek, Bellefonte, PA, USA). For analysis, approximately 900 mg of ground and homogenized sample was weighed into a 20 mL headspace vial, sealed, and incubated at 70 °C for 30 min. Volatile compounds present in the headspace were extracted using a 75 µm CAR/PDMS fused silica fiber (23 Ga, Supelco, Bellefonte, PA, USA) via solid phase microextraction (SPME), and thermally desorbed into the GC injector port. The injector was maintained at 250 °C in splitless mode. The column temperature program was as follows: initially held at 45 °C for 2 min, ramped to 72 °C at 2 °C/min and held for 2 min, then increased to 78 °C at 2 °C/min, followed by 85 °C at 1.5 °C/min, 105 °C at 10 °C/min, 137 °C at 1.5 °C/min, 157 °C at 5 °C/min, and finally to 240 °C at 20 °C/min with a final hold for 3 min. Helium (>99.999%) was used as the carrier gas at a constant flow rate of 1.0 mL/min. The mass spectrometer operated in electron ionization (EI) mode at 70 eV with a scanning range of *m*/*z* 30–650. The interface, ion source, and quadrupole temperatures were set at 250 °C, 230 °C, and 150 °C, respectively. Identification of volatile compounds was performed by comparison with authentic standards, retention indices, and mass spectral libraries (FFNSC 1.2 and Wiley 7). Retention indexes were calculated using a C7–C20 n-alkanes series (1000 mg/L in n-hexane) from Sigma-Aldrich. At the time of injection, the fiber was kept in the injection port for 5 min at 250 °C to allow complete desorption of all compounds. The ratios of detected compounds were calculated by dividing the tail-to-tail peak area of each component by the total area.

### 2.5. Cell Culture and Cytotoxicity Assays

Cell culture studies were carried out with minor modifications to the method of Gülüm et al. [47]. MCF-7 breast cancer cells, obtained from the American Type Culture Collection (ATCC), were cultured in Dulbecco’s modified Eagle’s medium (DMEM) (EuroClone, Via Figino, Italy) supplemented with 10% fetal bovine serum (FBS) and 1% glutamine. The cells were then incubated at 37 °C in a humidified atmosphere containing 5% CO_2_. Once the cells reached 80–90% confluence, they were detached with 0.25% trypsin-EDTA and seeded at appropriate densities (5000–10,000 cells per well) into 96-well plates for treatment. *G. lucidum* plant extracts were applied at various concentrations serially diluted from 1000 mg/L and incubated for either 48 or 72 h to assess their cytotoxic effects. Control wells received the corresponding solvents (e.g., methanol for methanol extracts) at equivalent final concentrations. All experiments were performed in duplicate, with each treatment condition assessed in at least four wells per experiment. Cell viability was determined using the MTT assay, with absorbance measured at 570 nm. IC_50_ values were calculated using GraphPad Prism software (version 8.0.2).

### 2.6. Metabolic Pathway Modulation in Cancer Cells

Gene expression data from *G. lucidum* methanol-treated cells were analyzed using the Reactome database. Only interactions with a *p*-value ≤ 0.05 were considered to identify significantly enriched biological pathways. A hypergeometric test was applied for pathway enrichment, and the Benjamini–Hochberg method was used to correct for multiple testing. A false discovery rate (FDR) threshold of 0.05 was set to reduce false positives and ensure statistical reliability. The Reactome database can be found here: https://reactome.org.

### 2.7. Flow Cytometry Analyses

#### 2.7.1. Cell Cycle Distribution (PI Staining) Assay

Cell cycle analysis was performed using the Sigma-Aldrich Mak344 Cell Cycle Analysis kit, following the specified protocol. All cell cycle analyses were performed in triplicate to ensure reproducibility. MCF-7 cells were treated at IC_50_ concentrations for 96 h, then washed with PBS and fixed using cold 70% ethanol. After fixation, the cells were washed with cell cycle assay buffer, and a staining solution containing enzyme A and nuclear dye was added, followed by a 30-min incubation in the dark. The stained cells were analyzed by flow cytometry to determine the phases of the cell cycle [53].

#### 2.7.2. Apoptosis (Annexin V FITC/PI)

Apoptosis in MCF-7 cells was evaluated using the ApopNexin^TM^ FITC Annexin-V/PI Apoptosis Detection Kit (Merck, Darmstadt, Germany) after 96 h of treatment with IC_50_ concentrations in triplicate. The cells were harvested, washed, and stained with FITC-labeled Annexin-V and propidium iodide, following incubation in a binding buffer. After a 15-min incubation in the dark at room temperature, the samples were analyzed by flow cytometry to identify apoptotic and necrotic cell populations [47].

### 2.8. Gene Expression Profiling

RNA extraction, cDNA synthesis, and gene expression analysis were conducted to evaluate approximately 46 genes involved in various cancer signaling pathways. Total RNA was isolated from tumor samples using the innuPREP RNA Mini Kit 2.0 (Innuscreen GmbH, Berlin, Germany). The concentration and purity of RNA were measured spectrophotometrically using an Eppendorf BioSpectrometer^®^ (Eppendorf, Hamburg, Germany), with results expressed in ng/μL and A260/280 ratios. Complementary DNA (cDNA) was synthesized using the SensiFAST™ cDNA Synthesis Kit (Bioline, Memphis, Tennessee, USA). Quantitative real-time PCR (RT-qPCR) was performed using the Analytical Jena qTOWER3 system (Analytik Jena, Jena, Germany), with SYBR Green Master Mix (EuroClone, Pero, Italy) and GAPDH as the reference gene. All RT-qPCR reactions were performed in duplicate to ensure reproducibility. Data were analyzed according to the manufacturer’s protocol, and relative gene expression levels were calculated using the 2^−ΔΔCt^ method [47]. This approach provided insights into the potential mechanisms of action for each treatment, based on changes in gene expression profiles.

### 2.9. Statistical Analysis

The extraction study was designed using a factorial experimental approach with three replications and analyzed using JMP Pro 16 software (SAS, Cary, NC, USA). The data collected underwent Levene’s test for homogeneity and a three-way analysis of variance (ANOVA) to assess the significance of the main effects and interactions between the variables, which included different types of mushrooms, treatment time and solvents. Fisher’s Least Significant Difference (LSD) test was applied at a significance level of α = 0.05 to identify statistically significant differences among the group means. Additionally, cell viability analyses were performed using GraphPad Prism version 8.0.2.

## 3. Results

### 3.1. Phytochemical and Antioxidant Profiles

#### 3.1.1. Total Phenolics, Flavonoids, and Anthocyanins

Table 1 summarizes certain biochemical properties of *G. lucidum* extracts using ethanol and methanol. TPC was slightly lower in the methanolic extract, measuring 3.54 mM, than in the ethanolic extract, 8.21 mM. Similarly, the TFC was higher in the ethanolic extract at 3.31 mM than in the methanolic extract at 1.16 mM. The anthocyanin content was significantly higher in the ethanol extract, reaching 4.53 mM; the methanolic extract contained only 0.59 mM.

#### 3.1.2. Soluble Proteins and Carbohydrates

The analysis revealed significant differences in the total protein and carbohydrate content of *G. lucidum* extracts depending on the solvent used (Table 2). The ethanol extract had the highest total protein content, at 14.11 ± 0.59%, which was significantly higher than the methanol extract’s content of 7.40 ± 0.31% (*p* < 0.05). Similarly, the ethanol extract had a notably higher total carbohydrate content, at 76.21 ± 3.18%, compared to the methanol extract’s content of 36.70 ± 1.53% (*p* < 0.05).

#### 3.1.3. Total Antioxidant Capacity

The antioxidant activity of *G. lucidum* extracts varied significantly depending on the solvent used for extraction. The ethanol extract demonstrated significantly higher antioxidant capacity in all four assays compared to the methanol extract (*p* < 0.05). The ethanol extract exhibited DPPH radical scavenging activity of 23.02 ± 0.96 mM, CUPRAC reducing power of 95.98 ± 4.01 mM, ABTS scavenging activity of 42.59 ± 1.78 mM, and a FRAP value of 35.37 ± 1.48 mM at 593 nm. In contrast, the methanol extract showed lower values across all tests: 9.93 ± 0.41 mM for DPPH, 30.32 ± 1.27 mM for CUPRAC, 18.15 ± 0.76 mM for ABTS, and 15.09 ± 0.63 mM for FRAP, respectively (Table 3).

### 3.2. Phenolic Compound Profiling

The phenolic and organic acid profiles of *G. lucidum* extracts using ethanol and methanol revealed notable differences (Table 4). The methanol extract was particularly rich in gallic acid (123.18 µg/mL), 4-hydroxybenzoic acid (67.31 µg/mL), and (−)-epicatechin (226.70 µg/mL), while these compounds were either present in lower concentrations or not detected at all in the ethanol extract. Conversely, the ethanol extract demonstrated a significantly higher presence of syringic acid (122.65 µg/mL), syringrin hydrate (216.49 µg/mL), vanillic acid (340.44 µg/mL), and the combination of ferulic and trans-ferulic acids (279.72 µg/mL), which were either absent or found in much lower amounts in the methanol extract. Furthermore, caffeic acid (30.84 µg/mL), procatechin (137.58 µg/mL), and the combination of sinapic and trans-sinapic acids (92.20 µg/mL) were more abundant in the ethanol extract, whereas the methanol extract contained slightly lower levels of these compounds. Notably, succinic acid was found at an exceptionally high concentration in the ethanol extract (1900.41 µg/mL), compared to only 63.81 µg/mL in the methanol extract, indicating a solvent-specific efficiency for extracting certain organic acids. Interestingly, compounds such as quercitrin (63.10 µg/mL) and (−)-epicatechin were detected exclusively in the methanol extract. In contrast, syringin hydrate and several other compounds were unique to the ethanol extract. This suggests a significant difference in the polarity and extractability of compounds between the two solvents used.

### 3.3. Effects of Volatile Compounds

The volatile compound profile of *G. lucidum* reveals its diverse chemical composition, which includes aldehydes, carboxylic acids, esters, alcohols, ketones, hydrocarbons, and heterocyclic compounds.

The volatile compound profile of *G. lucidum* reveals its diverse chemical composition, which includes aldehydes, carboxylic acids, esters, alcohols, ketones, hydrocarbons, and heterocyclic compounds (Table 5). The total ion chromatogram shows that aldehydes are the most dominant group, making up about 47.84% of the total area. Hexanal is the most prominent aldehyde, making up 21.67% of the total, followed by nonanal at 6.37%, benzaldehyde at 5.02%, and octanal at 3.44%. These compounds are typically associated with green, fatty, and floral odors. They may result from lipid oxidation processes and contribute to aroma and potential biological activity [54,55,56,57]. The second most abundant chemical group was carboxylic acids and esters, which made up 21.75% of the total. The most prevalent compound in this group was acetic acid, accounting for 13.99% of the total. Acetic acid is a common fermentation product known for its volatile acidity and antimicrobial properties. Additionally, 2-pyridinepropanoic acid ethyl ester was detected as 6.03%. Alcohols and ketones comprised a significant portion of the volatile compounds, contributing 15.42%. The most abundant representatives of these subgroups were 1-pentanol (3.58%) and 6-methyl-5-hepten-2-one (4.67%). These compounds are typically associated with fruity, floral, and slightly earthy aromas, and they may also serve as intermediates in secondary metabolism [58,59,60,61,62,63].

Additionally, hydrocarbons and other miscellaneous compounds accounted for 12.23% of the volatile content. This category includes saturated hydrocarbons such as tetradecane (2.87%) and dodecane (1.47%), which contribute less to the aroma, but may affect the extract’s physical properties. Other detected compounds included furans (e.g., 2-amyl furan at 3.08%), dimethyl formamide (1.01%), and minor aromatic or cyclic compounds, such as 1,4-epoxycyclohex-2-ene (0.98%).

### 3.4. Cell Culture and Cytotoxicity

**Figure 2:**
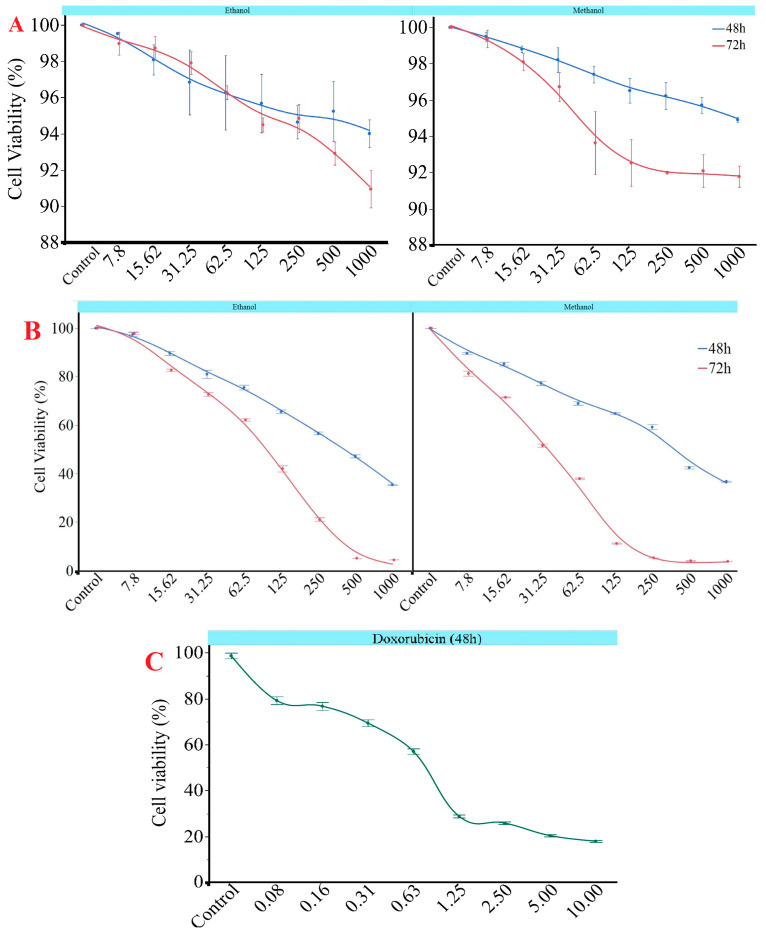
Impact of *G. lucidum* concentrations (µg/mL) according to different solvents on cell viability in (**A**) MCF-10A and (**B**) MCF-7 cell line at 48 and 72 h and (**C**) doxorubicin in MCF-7 cell lines at 48 h (µM). All experiments were performed in duplicate.

The results revealed a significant, concentration- and time-dependent decrease in cell viability for both extracts (*p* < 0.05). Notably, the methanol extract exhibited significantly higher cytotoxicity at most concentrations. At the lowest concentration (7.8 µg/mL), cell viability was largely maintained in both extracts after 48 h, remaining above 96%. However, after 72 h, a statistically significant reduction in viability was observed for the methanol extract, decreasing from 89.61% at 48 h to 81.31% (*p* < 0.05), indicating early cytotoxic potential. At 62.5 µg/mL, the methanol extract reduced viability to 37.99% after 72 h (Figure 2). This value was significantly lower than the 48-h value (68.91%) and the ethanol extract value at the same time point (62.20%) (*p* < 0.05). Similarly, at 125 µg/mL, the methanol extract caused a dramatic decrease in viability, reducing it to 11.17% at 72 h. This was significantly lower than the ethanol extract’s viability at the same time point (42.07%) (*p* < 0.05), which further confirms the methanol extract’s stronger cytotoxic profile. At a concentration of 250 µg/mL, the methanol extract significantly reduced cell viability to 5.29%. This is notably lower than the 21.07% viability observed for the ethanol extract at the same time point (*p* < 0.05). The maximum cytotoxic effect was observed after 72 h at a concentration of 500 µg/mL: viability was 4.04% for the methanol extract and 5.11% for the ethanol extract. Both values were significantly lower than their respective 48-h measurements (*p* < 0.05). These results suggest that *G. lucidum* extracts exhibit time- and dose-dependent cytotoxicity, with the methanol extract demonstrating significantly greater effects at various concentrations and time points. The methanolic and ethanolic extracts of *G. lucidum* demonstrated more than 90% cell viability in MCF-10A healthy breast cells after 48 and 72 h of incubation. At lower concentrations, cell viability remained in the range of 97% to 100%, with only slight reductions noted at higher concentrations. Similar to *G. lucidum* treatments, cell viability decreased in a dose-dependent manner with increasing doxorubicin concentration. While the cell viability was 79.21% at 0.078 μM doxorubicin, a progressive decline was observed, decreasing to 17.95% at 10 μM treatment (Figure 2).

The findings were reinforced by the IC_50_ values shown in Table 6, which significantly decreased from 48 to 72 h for both extracts (*p* < 0.05). At 72 h, the methanol extract demonstrated a considerably lower IC_50_ value of 62.37 ± 18.81 µg/mL compared to the ethanol extract, which had an IC_50_ of 116.91 ± 11.72 µg/mL. This indicates that the methanol extract exhibited stronger cytotoxic potency over time. The IC_50_ values for both methanolic and ethanolic extracts of *G. lucidum* on MCF-10A healthy breast cells did not fall within the tested concentration range, remaining above 1000 µg/mL. This suggests that cell viability remained consistently above 90% even at the maximum dosage applied. Furthermore, doxorubicin demonstrated an IC_50_ value of 0.36 µg/mL 48 h after treatment.

A three-way ANOVA revealed that solvent type, treatment duration, and extract concentration significantly influenced cell viability (*p* < 0.05) (Table 7). Concentration had the most substantial impact among these factors (F = 7851.71), followed by the time after treatment duration (F = 11139.01) and solvent type (F = 1352.58). Significant interactions were also found among all factor combinations, including time × concentration, solvent × concentration, and solvent × time × concentration (*p* < 0.05). These results suggest that cytotoxic effects depend on both individual factors and their combinations.

### 3.5. Flow Cytometry Analyses

#### 3.5.1. Cell Cycle Distribution (PI Staining)

**Figure 3:**
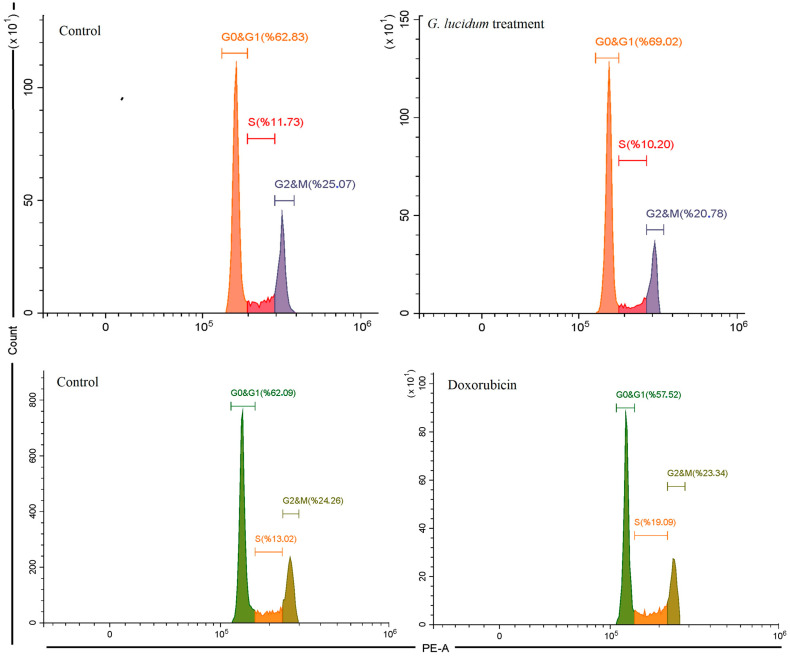
The cell cycle analysis of MCF-7 cancer cells under *G. Lucidum*-Methanol and doxorubicin treatments.

Flow cytometry analysis of DNA content indicated that treatment with *G. lucidum* methanol extract led to changes in the cell cycle distribution of MCF-7 cells (Figure 3). In the control group, 63.83% of the cells were in the G0/G1 phase, 11.73% in the S phase, and 25.07% in the G2/M phase. After treatment with the *G. lucidum* methanol extract, there was a significant increase in the G0/G1 population, rising to 69.02%. Additionally, the percentages of cells in the S phase decreased to 10.2% and in the G2/M phase to 20.78% compared to the control group. These findings suggest that the methanol extract of *G. lucidum* may inhibit cell cycle progression by causing an arrest in the G0/G1 phase, which could contribute to reduced cell proliferation and the induction of apoptosis. Doxorubicin induced cell cycle arrest in the S phase, increasing from 13.02% to 19.09%, while reducing the G0&G1 phase and showing a slight decrease in the G2&M phase (Figure 3).

#### 3.5.2. Apoptotic Induction-Apoptosis (Annexin V FITC/PI)

**Figure 4:**
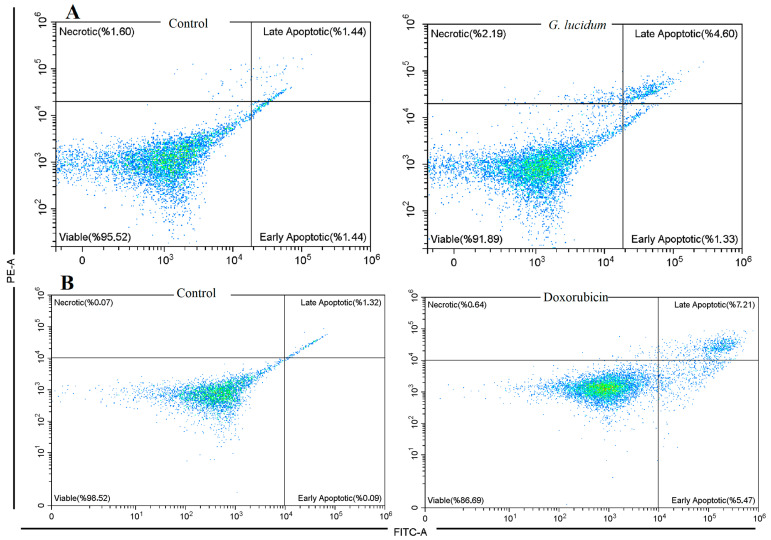
Apoptosis in MCF-7 cancer cells by the treatment of *G. Lucidum*-Methanol (**A**) and doxorubicin (**B**).

Flow cytometric analysis using Annexin V-FITC/PI staining showed significant differences in cell death patterns between the treated and control groups (Figure 4). In the control group, 95.52% of MCF-7 cells remained viable, with minimal early apoptotic cells (1.44%), late apoptotic cells (1.44%), and necrotic cells (1.6%). In contrast, treatment with *G. lucidum* methanol extract reduced the percentage of viable cells to 91.89%. This treatment also increased the proportion of late apoptotic cells to 4.6%, suggesting the induction of apoptosis. The percentage of early apoptotic cells remained relatively unchanged at 1.33%, while necrotic cells slightly increased to 2.19%. These findings indicate that the *G. lucidum* methanol extract exerts its cytotoxic effects primarily by promoting late-stage apoptosis in MCF-7 cells. Doxorubicin, on the other hand, exhibited a slightly higher apoptosis in both early and late phases at 5.47% and 7.21%, respectively, with a total of 86.69% viable cell ratio. Moreover, the necrotic cell ratio was slightly lower than *G. lucidum* treatment, with 0.64% necrotic cells (Figure 4). The results demonstrated that both *G. lucidum* and doxorubicin induce apoptosis more than necrosis.

### 3.6. Modulation of Cancer Metabolism Genes

**Figure 5:**
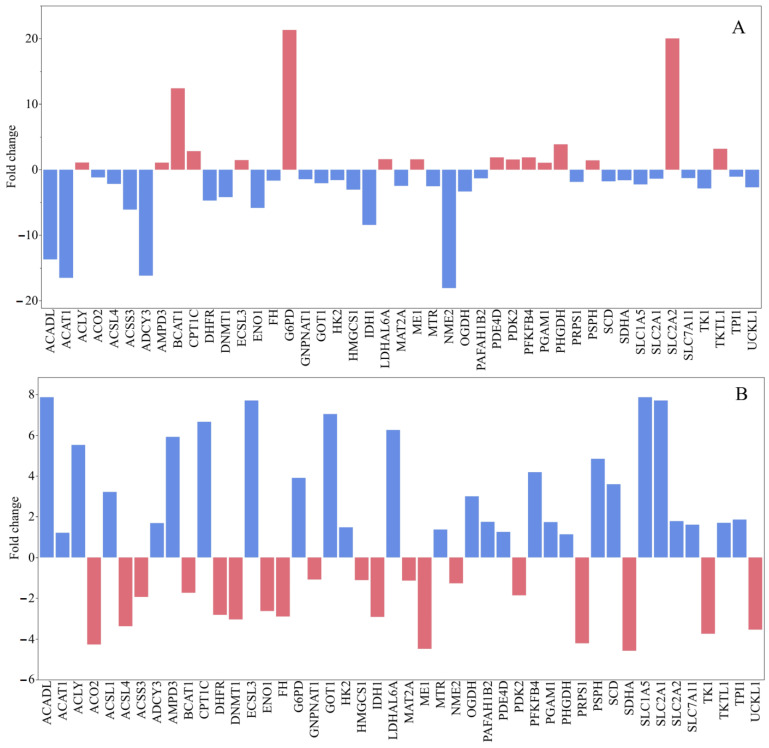
Gene expression levels in MCF-7 human breast cancer cells by the induction of *G. lucidum*-Methanol extract (**A**) and doxorubicin (**B**).

Upregulated Metabolic Genes: Among the significantly upregulated metabolic genes, *BCAT1* exhibited a remarkable fold increase of +12.42, indicating a strong activation of branched-chain amino acid catabolism. Following this, *SLC2A2*, which encodes the glucose transporter *GLUT2*, showed an increase of +20.04, suggesting increased glucose influx into cells and a potential metabolic shift toward glycolysis. Furthermore, *G6PD*, a key enzyme in the pentose phosphate pathway, was highly upregulated with a value of +21.33, reflecting enhanced NADPH generation and improved redox balance. Moderate increases were observed in *PHGDH* (+3.88), involved in serine biosynthesis; *TKTL1* (+3.19), associated with non-oxidative glycolysis; and *CPT1C* (+2.84), related to the transport of fatty acids into mitochondria. Several other genes, such as *PSPH*, *LDHAL6A*, *PDK2*, *ECSL3*, *ME1*, and *PGAM1*, demonstrated mild increases (ranging from +1.0 to +1.6), pointing to a broader metabolic reorganization to meet biosynthetic and energetic requirements (Figure 5A).

Downregulated Metabolic Genes: Conversely, a significant downregulation was observed in *ACAT1* (−16.51), indicating a severe inhibition of acetyl-CoA metabolism and cholesterol homeostasis. *NME2* (−18.06) and *ADCY3* (−16.17), both involved in nucleotide metabolism and cyclic AMP signaling, respectively, were also sharply downregulated. Additionally, *ACADL* (−13.69) and *IDH1* (−8.43) showed notable decreases, suggesting impaired fatty acid β-oxidation and disrupted flux in the TCA cycle. The glycolytic enzyme *ENO1* experienced a decline of −5.84, while *ACSS3*, a regulator of acetate metabolism, decreased by −6.08. Moderate reductions were also noted in *DHFR*, *DNMT1*, *SLC1A5*, and *MAT2A*, with changes ranging from −4 to −2 (Figure 5A). These suggest repression of folate metabolism, DNA methylation, amino acid transport, and methylation cycles. Collectively, these patterns indicate a reorganization of the metabolic network toward a bias for glycolysis and biosynthesis, while oxidative phosphorylation and mitochondrial metabolism appear to be downregulated.

Doxorubicin treatment of MCF-7 human breast cancer cells elicited notable changes in the expression of a panel of metabolic and regulatory genes. Several genes involved in lipid metabolism and mitochondrial fatty acid oxidation were strongly upregulated, including *ACADL*, *GNPNAT1*, *GOT1*, *ECSL3*, *SLC1A5*, *LDHAL6A*, *CPT1C*, and *SLC2A1*, suggesting a shift toward enhanced lipid utilization and glucose uptake under chemotherapeutic stress. Conversely, several genes associated with nucleotide biosynthesis and epigenetic regulation were downregulated, such as *PRPS1*, *TK1*, *DNMT1*, *IDH1*, *SDHA*, and *ME1*, consistent with reduced proliferative capacity and diminished one-carbon and nucleotide synthesis pathways. The results also showed upregulation of components linked to the oxidative stress response and the pentose phosphate pathway, exemplified by *G6PD* and *PSPH*, indicating an increased capacity to generate NADPH to counteract doxorubicin-induced reactive oxygen species (Figure 5B).

## 4. Discussion

Although the ethanol extract of *G. lucidum* showed higher levels of total phenolics and flavonoids, along with strong antioxidant activity, our findings indicated that the methanol extract exhibited greater cytotoxicity against MCF-7 breast cancer cells. This discrepancy can be attributed to differences in how various solvents extract bioactive compounds. Previous studies have demonstrated that methanol extracts of *G. lucidum* are particularly rich in lycopene and β-carotene [64], both of which are known to promote cytotoxic and pro-apoptotic effects in cancer cells [65]. Furthermore, the hydroxyl radical scavenging activity was more pronounced in the methanol extract, potentially enhancing its ability to induce oxidative stress-related cytotoxic effects. Therefore, the stronger anticancer activity of the methanol extract is likely linked not only to its specific phytochemical profile but also to the functional properties of individual compounds that work together to modulate cellular responses [66]. Moreover, other bioactive compounds, such as polysaccharides, carotenoids, amino acids, and secondary metabolites, may also contribute to the observed antioxidant activity [67].

Several studies demonstrate that extracts from *G. lucidum* exert cytotoxic effects on MCF-7 cells in a dose-dependent manner. The ethanolic extract of *G. lucidum* has been reported to inhibit cell proliferation, with IC_50_ values indicating significant antiproliferative activity [68,69]. The ability of *G. lucidum* to induce apoptosis in MCF-7 cells is supported by evidence of DNA damage and increased expression of apoptosis markers such as *γ-H2AX* [70]. Moreover, these effects include cell cycle arrest at the G1 phase, facilitated by upregulation of tumor-suppressing proteins such as *p21* and downregulation of cyclin D1, demonstrating biological activity against MCF-7 cells [71,72]. In this study, we also observed significant cytotoxic effects of *G. lucidum* in both ethanol and methanol extracts, while methanol extract exerted a higher cytotoxicity. The results suggest that *G. lucidum* extracts are not significantly toxic to MCF-10A cells, indicating a safe profile for healthy cells. These findings support the notion that the potential therapeutic effects of the extracts may be more targeted towards cancer cells while being well tolerated by normal cells. This study found that when *G. lucidum* extract was applied to MCF-7 breast cancer cells, it exhibited multidimensional suppressive effects on energy metabolism, the cell cycle, and programmed cell death. The combined action of these three mechanisms enhances the extract’s potential efficacy as an anticancer agent. In the analysis of the cell cycle, it was found that the cells accumulated in the G0/G1 phase after the application of the extract. This indicates that the cells were unable to proceed to DNA synthesis and therefore could not divide. According to the literature, components of *G. lucidum* achieve this effect by decreasing levels of Cyclin D1 and *CDK4*/*6*, while increasing levels of *p21* and *p27* [73].

**Figure 6:**
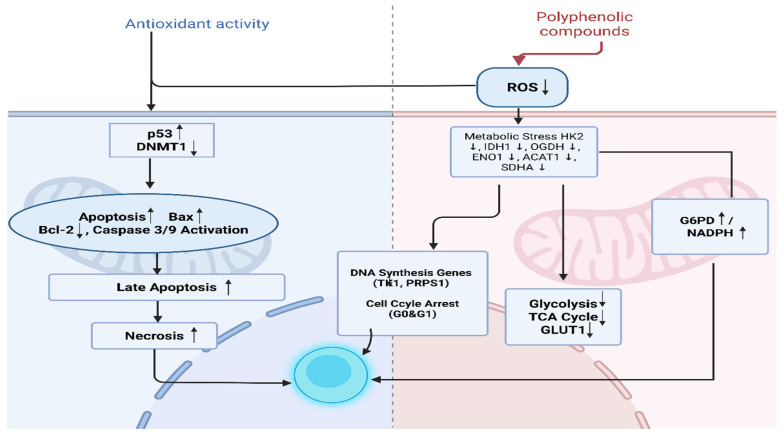
The Effect of polyphenolic compounds on cellular metabolism and apoptosis. Created in BioRender. TUTAR, Y. (2025) https://BioRender.com/spybjcy. Access date: 12 June 2025. The up and down arrows indicate upregulation and downregulation, respectively.

In flow cytometry-based analyses of apoptosis, a significant increase was observed in the percentage of late apoptotic and necrotic cells. This finding indicates that *G. lucidum* extract activates the mitochondrial apoptosis pathway and leads some cells to undergo unprogrammed cell death. Literature reports indicate that compounds such as (−)-epicatechin [74], gallic acid [75], ferulic acid [21], and caffeic acid [24] accelerate this process by influencing Bcl-2, Bax, and Caspase-9 levels. Specifically, they decrease Bcl-2 and increase Bax and Caspase-9 activity (Figure 6).

Components such as triterpenoids and polysaccharides isolated from *G. lucidum* have been implicated in its cytotoxic effects. These bioactive compounds contribute significantly to its anticancer properties, impacting immune responses and inflammation [76]. Additionally, *G. lucidum* extracts interact with oxidative stress pathways, suggesting potential therapeutic benefits in mitigating cancer cell migratory behaviors exacerbated by oxidative conditions [77].

Analyzing phenolic content is crucial for understanding the biological effects of the extract. The methanol extract was found to be rich in several compounds, including gallic acid (123.18 µg/mL), 4-hydroxybenzoic acid (67.31 µg/mL), and notably, (−)-epicatechin (226.70 µg/mL). These compounds are recognized for their antioxidant, anti-inflammatory, and pro-apoptotic properties in the literature [78,79].

In the ethanol extract, high concentrations of vanillic acid (340.44 µg/mL), ferulic acid, and syringic acid were identified. Additionally, the significantly high level of succinic acid (1900.41 µg/mL) may contribute to increased cellular stress and acidosis, as it is an organic acid directly involved in energy metabolism. These varying compound profiles indicate that the two extracts may influence cells in different ways, yet both demonstrate cancer-suppressive effects. Phenolic compounds found in mushroom extracts, such as gallic acid, 4-hydroxybenzoic acid, (−)-epicatechin, vanillic acid, ferulic acid, and syringic acid, are recognized for their antioxidant and anticancer properties. Gallic acid and 4-hydroxybenzoic acid promote apoptosis and inhibit the proliferation of cancer cells by modulating oxidative stress [80,81]. (−)-Epicatechin plays a role in antioxidant defense and regulation of apoptosis, while vanillic acid helps inhibit tumor growth by modulating the Raf/MEK/ERK pathway [82,83]. Ferulic acid has been shown to suppress metastasis and promote autophagy as well as cell cycle arrest [22,84]. Additionally, syringic acid enhances the synergistic antioxidant and cytotoxic effects of mushroom extracts [84]. Together, these phenolic compounds enhance the overall anticancer efficacy of bioactive mixtures derived from mushrooms.

The GC-MS analysis revealed that *G. lucidum* contains a high proportion of aldehydes (47.84%), the dominant contributors of which are hexanal (21.67%), nonanal (6.37%), and phenylacetaldehyde (2.06%). These aldehydes impart green, fatty, and floral aroma notes and are known for their pro-oxidant and cytotoxic properties. Hexanal, a major lipid peroxidation product, can exacerbate oxidative stress and trigger mitochondria-dependent apoptosis in cancer cells. These compounds contribute to redox imbalance and G0&G1 phase arrest by downregulating important proliferation-related genes such as *TK1*, *PRPS1*, and *DHFR* [85,86]. Several volatile organic compounds found in mushroom extracts—such as hexanal, nonanal, benzaldehyde, 1-pentanol (amyl alcohol), 6-methyl-5-hepten-2-one, acetic acid, and 2-pyridinepropanoic acid ethyl ester—exhibit significant biological properties that may be linked to anticancer activity. Hexanal and nonanal, which are aldehydes present in Tricholoma matsutake, contribute to the mushroom’s aroma and may have mild cytotoxic or antibacterial effects that are relevant to cancer modulation [87,88]. Benzaldehyde shows stronger evidence of anticancer properties by inducing apoptosis and oxidative stress in cancer cells [36,52] 1-Pentanol and 6-methyl-5-hepten-2-one have been associated with inhibitory and antioxidant effects, although their direct mechanisms of action against cancer remain unclear [31,33]. Additionally, acetic acid promotes apoptosis and may enhance the effectiveness of cancer treatments [89,90]. Meanwhile, 2-pyridinepropanoic acid ethyl ester shows α-glucosidase inhibition, suggesting a potential link to metabolic regulation in cancer [33]. These findings may indicate that volatile compounds derived from mushrooms possess promising bioactivities that warrant further investigation into their mechanisms of action.

The effects of *G. lucidum* on breast cancer cells can be explained by several mechanisms that modulate critical oncogenic signaling pathways. Research shows that *G. lucidum* downregulates estrogen receptors and NF-κB signaling, both of which are vital in MCF-7 cell proliferation and survival [91]. By disrupting these pathways, *G. lucidum* potentially enhances apoptosis while decreasing the migration and invasive capabilities of MCF-7 cells [77]. Its polysaccharides and triterpenes induce cell death by increasing the expression of pro-apoptotic proteins and suppressing anti-apoptotic proteins. Additionally, *G. lucidum* reduces cell viability by influencing signaling pathways, such as the Akt/NF-κB pathway [92]. The mushroom also impacts inflammation by inhibiting the proliferation of treatment-resistant cancer cells. It accomplishes this by reducing levels of inflammatory cytokines, such as IL-6 and IL-8 [93]. Furthermore, *G. lucidum* modulates the immune system, enhancing T lymphocyte activity and bolstering the immune response against tumors [94]. *G. lucidum* may also help decrease drug resistance by increasing sensitivity to chemotherapy drugs, indicating its potential as a supportive treatment [92,95]. In this study, we observed the downregulations of key energy metabolism genes, *IDH1* (−8.43), *OGDH* (−3.33), *ACO2* (−1.18), *FH* (−1.66), *SDHA* (−1.61), indicating that TCA cycle genes were generally suppressed, suggesting a decrease in mitochondrial energy production. This implies that cells reduce mitochondrial phosphorylation and switch to alternative energy pathways that may result in the cells becoming less reliant on the TCA cycle. These findings confirm the mitochondrial aspect of the Warburg effect. Tumor-associated mutations in *IDH1/2* result in the accumulation of the oncometabolite 2-hydroxyglutarate (2-HG). This, in turn, inhibits α-ketoglutarate-dependent dioxygenases, including TET and JmjC enzymes. Consequently, DNA and histone demethylation are blocked, which promotes tumorigenesis [96]. Significant *IDH1* downregulation by *G. lucidum* may alter cancer cell metabolism by reducing *2-HG* production [97].

The decrease in *ACAT1* (−16.51) suggests that cholesterol metabolism is being suppressed. In contrast, the increase in *BCAT1* (12.42) indicates heightened metabolism of branched-chain amino acids. Additionally, the reduction in *ACADL* (−13.69) points to suppressed fatty acid oxidation. These data indicate that MCF-7 cells seek alternative carbon sources, such as Branched-Chain Amino Acids (BCAA) and Pentose Phosphate Pathway (PPP), to fulfill their energy needs rather than relying on fatty acid oxidation. Targeting metabolic pathways, such as the PPP or BCAA metabolism, may offer a new strategy for overcoming resistance in breast cancers, including the use of MCF-7 cell models. Interventions aimed at disrupting these pathways could increase the effectiveness of conventional treatments by making cancer cells more susceptible to drugs while reducing tumour viability [98]. Conversely, the increase in *CPT1C* (2.84) may enhance the transport of fatty acids into mitochondria. Furthermore, the decrease in *ACSL4* (−2.17) could contribute to a reduction in lipid metabolism, while the decline in *HMGCS1* (−3.04) may suggest decreased cholesterol biosynthesis. Notably, the substantial rise in *BCAT1* implies that MCF-7 cells may be aggressively converting BCAAs into energy and anabolic building blocks. Simultaneously, the suppression of genes such as *ACAT1* and *ACADL* could indicate a decline in traditional fatty acid metabolism. Conversely, several genes related to mitochondrial and fatty acid oxidation, such as *ACADL*, *CPT1C*, and *GNPNAT1*, were found to be upregulated by doxorubicin treatment. Oxidative stress response and pentose phosphate pathway components like *G6PD* are upregulated, aligning with increased need for NADPH to counteract doxorubicin-induced ROS. Oxidative instability is considered one of the crucial defense metabolisms in cancer cells [99]. The observations may indicate a compensatory shift in energy metabolism in response to doxorubicin and *G. lucidum* stress. This metabolic shift may provide a strong basis for targeted therapeutic strategies, such as the use of *G6PD* inhibitors and targeting *BCAT1*. Studies conducted by Barbieri et al. [93] highlight the anti-inflammatory and anticancer properties of mushroom extracts. Their findings suggest that *G. lucidum* may affect important metabolic pathways associated with cancer progression by targeting *G6PD* and *BCAT1*.

The reduction in DNMT1 expression may result in a relaxation of epigenetic regulations, contributing to alterations in gene expression patterns in cancer cells [100]. Studies have shown that silencing *DNMT1* inhibits the proliferation, metastasis, and invasion of esophageal squamous cell carcinoma (ESCC) cells. Further, in vivo experiments confirm that *DNMT1* silencing reduces tumor growth in mouse models. This effect is associated with cell cycle arrest at the G1 phase and apoptosis induction. The underlying mechanism involves demethylating and reactivating tumor suppressor genes, such as *RASSF1A* and *DAPK*. Therefore, *DNMT1* is a promising therapeutic target for ESCC treatment [101]. Furthermore, the decreased expression of cell signaling genes, such as *NME2* and *ADCY3*, could suggest a weakening of cAMP-mediated signaling, particularly the decline in *ADCY3* gene expression. *G. lucidum* extracts may inhibit the proliferation of cancer cells by targeting cAMP-regulated signaling pathways demonstrated that these extracts reduce integrin expression, thereby impairing cell adhesion and migration. This may enhance the mushroom’s anticancer effects. Compounds in *G. lucidum* may also reduce the activity of transcription factors (like NF-kB) that are activated via cAMP signaling pathways [102]. This reduced expression of growth factors and anti-apoptotic proteins. This regulatory effect can reduce breast cancer cell survival and inhibit tumor progression. Zhou et al. [103] study revealed that treatment of RPMI-8226 myeloma cells with *DNMT1* siRNAs led to a significant decrease in *DNMT1* expression at both the mRNA and protein levels. This downregulation of *DNMT1* expression inhibited cell growth, likely by causing an arrest at the G0/G1 phase of the cell cycle [103,104].

Decreases in genes such as *GOT1* and *MAT2A* suggest a suppression of various branches of amino acid metabolism. Pentose Phosphate Pathway (PPP) is vital for the production of NADPH and ribose-5-phosphate, which are necessary for cell survival and division. *TKTL1* is involved in the non-oxidative branch of the PPP and is often upregulated in rapidly proliferating cells, indicating that alternative pathways for nucleotide synthesis are activated as well. The increase in genes such as *PHGDH* and *PSPH*, which are enzymes in the serine biosynthesis pathway associated with the PPP, suggests that they play a role in nucleotide and amino acid synthesis. *PHGDH* is frequently amplified, especially in aggressive cancer types. Studies have shown that tumors with higher *PHGDH* expression can thrive in low-serine environments because they can efficiently convert glycolytic intermediates to serine [105]. *G. lucidum* contains bioactive compounds that may influence the expression of genes associated with serine metabolism, including *PHGDH*. Compounds from this fungus have been reported to affect signaling pathways, such as mTOR, which are integral to *PHGDH* regulation [106,107].

Cancer cells typically produce energy rapidly by consuming more glucose than normal cells. However, after applying the extract, it was observed that key glycolysis pathway enzymes, such as *HK2*, *ENO1*, and *TPI1*, were suppressed. This suggests that the cells are struggling to produce energy and are experiencing metabolic stress. Furthermore, the significant suppression of genes like *IDH1*, *OGDH*, and *SDHA* may indicate a decreased capacity for energy production through mitochondria in oxygenated environments [108]. This highlights the cells’ difficulty in generating even the energy required for survival. Notably, the substantial increase in the *G6PD* gene expression suggests that the cells are attempting to bolster their antioxidant defenses. This implies that the extract induces oxidative stress within the cells, prompting a defensive response. The significant increase in the *BCAT1* gene shows that the cells are turning to alternative energy sources but are still suppressed. Similarly, the significant downregulation of genes such as *ACAT1* and *ACADL*, which are involved in lipid metabolism regulation, may indicate that the cells are experiencing disruptions in crucial functions like membrane production, signal transmission, and energy storage [109]. Moreover, the decrease in *DNMT1* suggests that genetic control mechanisms are also being negatively affected, leading to a loss of cellular regulation. The interaction between *G6PD*, *DNMT1*, and *BCAT1* in tumors presents an opportunity to develop new treatment methods that target tumor metabolism. Inhibiting *G6PD* can disrupt cell survival, but the co-regulation of *DNMT1* and *BCAT1* may enhance treatment responses. These molecular targets indicate the potential for combined approaches in cancer therapy [110].

## 5. Conclusions

This study demonstrates that *G. lucidum* extract affects MCF-7 breast cancer cells in several significant ways. It induces serious metabolic stress, prevents cell proliferation by arresting cells in the G0/G1 phase, and triggers programmed cell death (apoptosis) along with cell destruction (necrosis). The cytotoxic effects of *G. lucidum* extract are a result of the synergistic action of various volatile compounds, particularly aldehydes, acids, and ketones, along with phenolic compounds. These compounds disrupt redox balance, damage membrane integrity, and interfere with cell cycle regulation. This multi-target bioactivity highlights its potential as a functional food ingredient or adjunctive therapy for cancer. The combination of these effects strongly highlights the anticancer potential of the extract. These findings suggest that further evaluation of the extract as a potential anticancer agent is warranted, and it should be tested in future in vivo or clinical studies.

## Figures and Tables

**Table 1:** Total phenolics, flavonoids, and anthocyanin contents of *G. lucidum* ethanol and methanol extracts (per gram).

Mushroom/Solvent	TPC (mM)	TFC (mM)	Tant (mM)
*G. lucidum* Ethanol	8.21 ± 0.34 ^a^	3.31 ± 0.14 ^a^	4.53 ± 0.19 ^a^
*G. lucidum* Methanol	3.54 ± 0.15 ^b^	1.16 ± 0.05 ^b^	0.59 ± 0.02 ^b^

Different letters in the same column indicate significant differences in *p* < 0.05 according to Fisher’s LSD test.

**Table 2:** Total protein and carbohydrate content of *G. lucidum* extracts depending on the solvent.

Mushroom/Solvent	Tprotein (%)	Tcarb (%)
*G. lucidum* Ethanol	14.11 ± 0.59 ^a^	76.21 ± 3.18 ^a^
*G. lucidum* Methanol	7.40 ± 0.31 ^b^	36.70 ± 1.53 ^b^

Different letters in the same column indicate significant differences in *p* < 0.05 according to Fisher’s LSD test. Tprotein: total protein content, Tcarb: total soluble carbohydrates content.

**Table 3:** Total antioxidant activities of *G. lucidum* extract different solvents.

Mushroom/Solvent	DPPH (mM)	CUPRAC (mM)	ABTS (mM)	FRAP (mM)
*G. lucidum* Ethanol	23.02 ± 0.96 ^a^	95.98 ± 4.01 ^a^	42.59 ± 1.78 ^a^	35.37 ± 1.48 ^a^
*G. lucidum* Methanol	9.93 ± 0.41 ^b^	30.32 ± 1.27 ^b^	18.15 ± 0.76 ^b^	15.09 ± 0.63 ^b^

Different letters in the same column indicate significant differences in *p* < 0.05 according to Fisher’s LSD test.

**Table 4:** The phenolic compounds detected in *G. lucidum* by HPLC.

Mushroom/Phenolic and Organic Acid Profiles	*G. lucidum* Ethanol (µg/g)	*G. lucidum* Methanol (µg/g)
Gallic Acid	18.81	123.18
4-Aminobenzoic Acid	n.d.	n.d.
Pro Catechin	137.58	112.16
Chlorogenic Acid	67.09	58.41
Syringic Acid	122.65	n.d.
4-Hydroxybenzoic Acid	44.58	67.31
Syringin Hydrate	216.49	n.d.
Caffeic Acid	30.84	26.86
Vanillic Acid	340.44	275.59
Ferulic Acid	279.72	122.81
Synapic Acid	92.20	119.05
Coumaric Acid	n.d.	n.d.
Rutintrihydrate	n.d.	n.d.
Quercitrin	n.d.	63.10
(−)-Epicatechin	n.d.	226.70
(+)-Catechin	n.d.	n.d.
Salicylic Acid	n.d.	n.d.
Succinic Acid	1900.41	63.81

n.d.—not detected.

**Table 5:** Volatile compounds detected in *G. lucidum* as a result of GC-MS analysis (%).

Group	Compound Name	% Area	RI
1. Aldehydes	Acetaldehyde (Ethanal)	2.37	691
	Butanal, 3-methyl-(3-Methylbutanal)	1.28	725
	Butanal, 2-methyl-(2-Methylbutanal)	0.66	729
	Pentanal	2.21	743
	Hexanal	21.67	816
	Octanal	3.44	1095
	Nonanal	6.37	1305
	Capraldehyde	1.06	1495
	Benzaldehyde	5.02	1027
	Phenylacetaldehyde	2.06	1169
	N-Heptanal	1.70	941
	Total	47.84	
2. Alcohols	Ethanol	1.46	694
	1-Pentanol (Amylol)	3.58	787
	1-Octen-3-ol	1.92	1059
	Total	6.96	
3. Ketones	2-Propanone (Acetone)	1.45	696
	3-Penten-2-one (E)	1.09	776
	6-Methyl-5-hepten-2-one	4.67	1070
	2-Heptenal (E)	0.59	1024
	2-Undecanone	0.66	1657
	Total	8.46	
4. Carboxylic Acids & Esters	Acetic acid	13.99	709
	Octanoic acid	0.63	927
	2-Pyridinepropanoic acid ethyl ester (1st)	6.03	688
	1,2-Benzenedicarboxylic acid, 3-nitro-	1.10	3019
	Total	21.75	
5. Aromatic Compounds	Benzene, 1,3-bis(1,1-dimethylethyl)-	1.72	1578
	Furfural	1.04	859
	Total	2.76	
6. Hydrocarbons (Alkanes)	Dodecane	1.47	1487
	Heptadecane	1.58	1631
	Tetradecane	2.87	1899
	Santalene (alpha)	1.24	1949
	Total	7.16	
7. Other Compounds	Formamide, N,N-dimethyl-	1.01	809
	Furan 2-amyl-	3.08	1077
	1,4-Epoxycyclohex-2-ene	0.98	1054
	Total	5.07	

**Table 6:** IC_50_ values of extracts from *G. lucidum* and doxorubicin (µg/mL).

Solvent/Moiety		48 h	72 h
*MCF-7 Cell Line*	*G. lucidum* Ethanol	469.81 ± 81.69 ^a^	116.91 ± 11.72 ^a^
	*G. lucidum* Methanol	408.12 ± 137.94 ^a^	62.37 ± 18.81 ^b^
*MCF-10A Cell Line*	*G. lucidum* Ethanol	>1000	
	*G. lucidum* Methanol	>1000	
Doxorubicin		0.0.36 ± 0.06	

Different letters in the same column indicate significant differences in *p* < 0.05 according to Fisher’s LSD test.

**Table 7:** Results of 3-way ANOVA on cell viability.

Variation Source	DF	Sum of Squares	F Ratio	*p*-Value
Solvent	1	1765.69	1352.58	2.06057 × 10^−48^
Time	1	14,541.18	11,139.01	1.12172 × 10^−80^
Concentration	8	81,998.71	7851.71	1.19 × 10^−102^
Time × Solvent	1	791.34	606.19	8.5256 × 10^−37^
Solvent × Concentration	8	947.27	90.71	1.73786 × 10^−34^
Time × Concentration	8	6546.74	626.88	2.36669 × 10^−63^
Solvent × Time × Concentration	8	705.82	67.58	2.11984 × 10^−30^

DF: degrees of freedom.

## Data Availability

The original contributions presented in this study are included in the article/Supplementary Material. Further inquiries can be directed to the corresponding author.

## Supplementary Materials

The following supporting information can be downloaded at https://www.mdpi.com/article/10.3390/antiox14121471/s1.

## Abbreviations

The following abbreviations are used in this manuscript:

*G. lucidum*	*Ganoderma lucidum*
ESCC	Esophageal squamous cell carcinoma
FRAP	Ferric reducing antioxidant power
CUPRAC	Cupric reducing antioxidant capacity
ABTS	2,2′-azino-bis(3ethylbenzothiazoline-6-sulfonic acid
DPPH	2,2-diphenyl-1-picrylhydrazyl
BCAA	Branched-Chain Amino Acids
PPP	Pentose Phosphate Pathway
MTT	[3-(4,5-dimethylthiazol-2-yl)-2,5-diphenyltetrazolium bromide]
PCR	Polymerase chain reaction
DMEM	Dulbecco’s modified eagle medium
EDTA	Ethylenediaminetetraacetic acid
DMSO	Dimethyl sulfoxide
