# Supplementary material for: Evaluation of Anticancer Potential of Ganoderma lucidum on MCF-7 Breast Cancer Cells Through Genetic Transcription of Energy Metabolism
Source: Antioxidants (Basel). 2025 Dec 8;14(12):1471. doi: 10.3390/antiox14121471 (PMC12730045; doi:10.3390/antiox14121471)
Supplement: Supplementary file 1 [file antioxidants-14-01471-s001.zip › antioxidants-3903234-supplementary.pdf]

Figure S1. HPLC chromatogram of 80 ppm standards of phenolic compounds examined in the study.

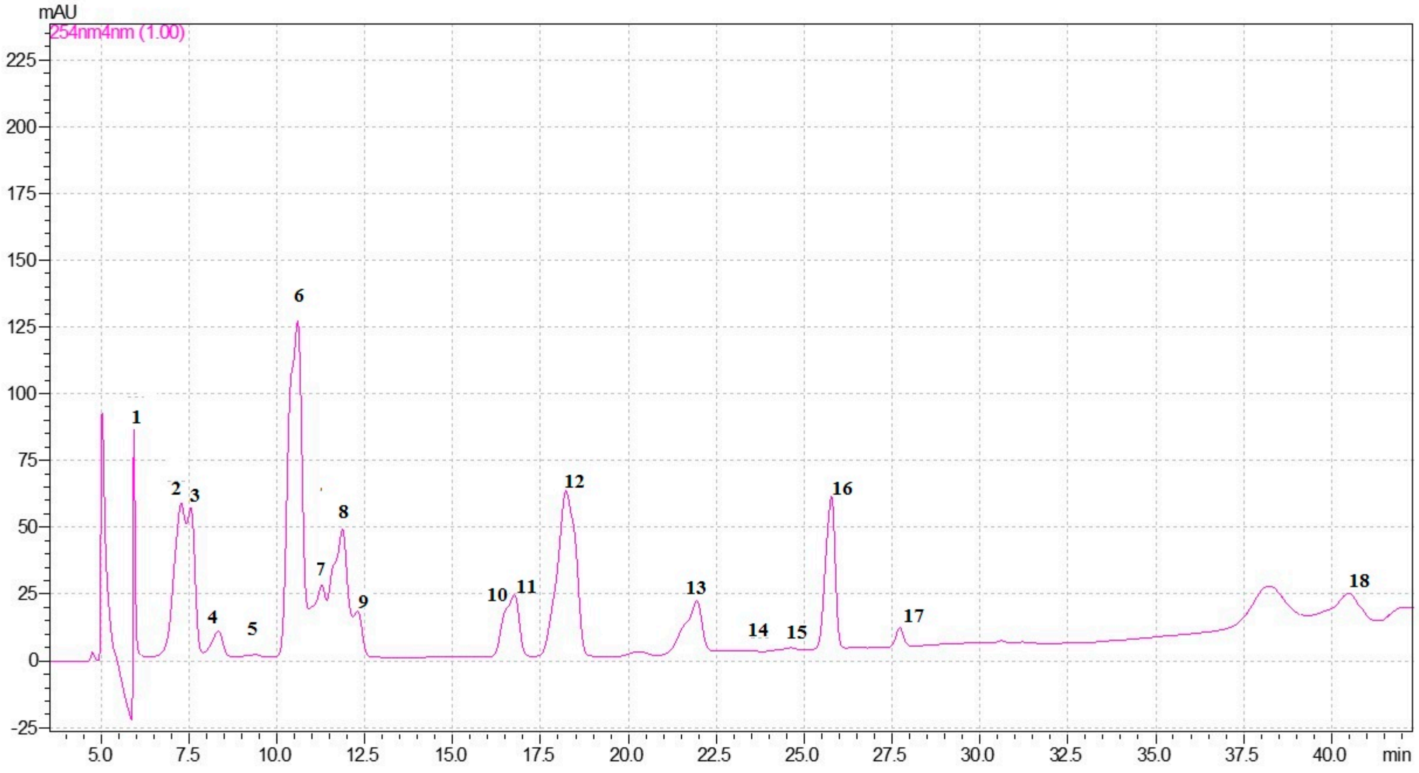

1)Gallic Acid, 2) 4-Aminobenzoic Acid, 3)Pro Catechin, 4)Chlorogenic Acid, 5)Syringic Acid, 6)4-Hydroxybenzoic Acid, 7)Syringin Hydrate, 8)Caffeic Acid, 9)Vanillic Acid, 10)Ferulic Acid, 11)Synapic Acid, 12)Coumaric Acid, 13)Rutintrihydrate, 14)Quercitrin, 15)(-)-Epicatechin, 16)(+)-Catechin, 17)Salicylic Acid, 18)Succinic Acid
